# Experimental and Theoretical Studies on Optical Properties of Tetra(Imidazole) of Palladium (II) Phthalocyanine

**DOI:** 10.3390/molecules27196151

**Published:** 2022-09-20

**Authors:** Abdelmajid Timoumi, Davoud Dastan, Bassem Jamoussi, Khaled Essalah, Omar Hammed Alsalmi, Noureddine Bouguila, Henda Abassi, Radhouane Chakroun, Zhicheng Shi, Ştefan Ţălu

**Affiliations:** 1 Department of Physics, Faculty of Applied Science, Umm AL-Qura University, Makkah 715, Saudi Arabia; 2 Department of Materials Science and Engineering, Cornell University, Ithaca, NY 14850, USA; 3 Department of Environmental Sciences, Faculty of Meteorology, Environment and Arid Land Agriculture, King Abdulaziz University, Jeddah 21589, Saudi Arabia; 4 Institut Préparatoire aux Etudes d’Ingénieurs d’El Manar, Unité de Recherche en Sciences Fondamentales et Didactique, Equipe de Chimie Théorique et Réactivité (UR14ES10), Université Tunis El Manar, Tunis 2092, Tunisia; 5 Laboratoire de Physique des Matériaux et des Nanomatériaux Appliquée à L’Environnement, Faculté des Sciences, Université de Gabès, Cité Erriadh, Zrig, Gabès 6072, Tunisia; 6 Laboratoire de Caracterisations, Applications et Modélisation de Matériaux, Faculte des Sciences de Tunis, Université Tunis El Manar, Campus Universitaire, Tunis 1068, Tunisia; 7 School of Materials Science and Engineering, Ocean University of China, Qingdao 266100, China; 8 The Directorate of Research, Development and Innovation Management (DMCDI), Technical University of Cluj-Napoca, Constantin Daicoviciu St., 400020 Cluj-Napoca, Romania

**Keywords:** PdPc(Im)_4_, band gap energy, DFT, atomic-scale simulation, thin films

## Abstract

In this work, the optical properties of tetra(imidazole) of palladium phthalocyanine (PdPc(Im)_4_) in solution form and thin films on glass and fluorine-doped tin oxide (FTO) substrates were investigated via the thermal evaporation technique. The optical band gap was evaluated by ultraviolet–visible spectroscopy (UV-Vis). The energy band gap values were determined based on the Tauc graph. In addition, time-dependent density functional theory (TD-DFT) was used to simulate the UV-Vis absorption spectrum of the (PdPc(Im)_4_) molecule in the Dimethyl Sulfoxide (DMSO) solution phase. A good correlation was found between the DFT results and the experimental optical results. The band gap values between the experimental and DFT-simulated values are presented. The energy band gap of (PdPc(Im)_4_) obtained from the DFT calculations showed that it can be efficiently regulated. Frontier molecular orbitals and molecular electrostatic potentials were also proposed in this work. The surface study of the layers deposited on FTO was considered by atomic force microscopy (AFM) and scanning electron microscopy (SEM), and the results demonstrated good homogeneity covering the entire surface. The SEM image showed a homogeneous distribution of the grains with some spherical or rod-shaped structures and no agglomeration structures. This work rendered a strategy for regulating the energy band gap and compared the experimental observations obtained with theoretical studies, which provides a fundamental insight into the optical band for optoelectronic and thin-film solar cells.

## 1. Introduction

Recently, numerous efforts have been dedicated to the production and study of several semiconductor materials in film form [1,2,3,4,5,6,7,8,9]. Metalophthalocyanines (MPcs) are the best significant organic materials that are extensively used in optoelectronic devices such as photoconducting agents [10], photovoltaic devices [11,12,13,14], nonlinear optics [15], electrocatalysis [5,16], solar cells [17,18], sensors [19,20,21,22,23,24,25,26,27], catalysis [5,28], and many others. Many researchers are interested in the study of various materials such as metal-substituted phthalocyanines (Pcs) [29]. Most of these materials are p-type organic semiconductors. There is considerable interest in the study of these materials because of their low cost, excellent thermal and chemical stability, and being versatile alternatives for the fabrication of thin-film-based devices. Moreover, PdPc exhibits longer exciton diffusion length in comparison with other bivalent metal phthalocyanines, such as ZnPc and CuPc [30]. This property, as well as the strong absorption within the visible spectral range, makes PdPc films a quite useful candidate for photovoltaic applications [31]. It has been assumed in the literature [32] that atmospheric oxygen absorbs at the air/MPc interface and at grain boundaries. It was reported that the formation of charge-transfer complexes by coordination of O_2_ to MPc at the air/phthalocyanine interface leads to the formation of oxidized MPc^+^ and O^2−^ species and injection of hole charge carriers into the film’s bulk [33].

However, there are limited studies on other phthalocyanines, particularly tetra(imidazole) of palladium phthalocyanines (PdPc(Im)_4_). The element palladium in PdPc(Im)_4_ has several useful applications [34,35,36]. Supported by this information, we report the optical study of PdPc(Im)_4_ that renders features for core system advantages for the applications in photovoltaic devices. In our previous work, we have studied PdPc in thin-film and pellet form [37,38]. A few other references have been reported on the fabrication and characterization of MPc thin films [39,40,41,42]. PdPc has been used for organic transistors [43,44], perovskite solar cells [45], and sensors [46,47,48]. Pcs are organic semiconductors with outstanding electrical features [49]. According to Lokesh et al. [50], the cyclic voltammetric data in DMSO showed that the central metal ion Pd does not undergo a redox process, and the redox behavior observed was mainly due to the macrocyclic ring reduction process. This result confirms the hypothesis of the works of Gould R. D [51] and de Haan A. [52] for the oxidation of the MPc species in MPc^+^ by atmospheric oxygen at the interface of air/MPc. The UV-Vis spectrum of phthalocyanine materials is at the origin of the molecular orbitals of the 18π aromatic electronic system [53]. These materials have been considered as electrophotographic materials due to their absorption capacity in the ultraviolet and visible range [54]. Currently, it has been reported that high mobility of PdPc(Im)_4_ thin films can be achieved by using high substrate heating temperature; the latter directly affects the crystal structure, morphology, and optical properties of the desired film.

The current study showed that the orientation of the grains strongly depends on the nature of the substrate, the thermal annealing temperature, and the deposition technique employed. The electronic structure, excitation process, and molecular interaction have been studied for certain metallic phthalocyanines [55] by the density functional theory (DFT) [56,57,58] and time-dependent DFT (TD) [59]. Herein, we studied the energy band gap of the palladium phthalocyaninein in DMSO solution and in thin-film forms using the UV-Vis absorption spectrum technique. Additionally, DFT calculations were used to calculate the E_g_ (gap) between molecular orbitals of the main peaks of IN spectra and the frontier highest occupied molecular orbital (HOMO) and lowest unoccupied molecular orbital (HOMO-LUMO). Optimized geometry for the PdPc(Im)_4_ molecule and other detailed quantitative information with electronic structure calculations are also given.

## 2. Experimental and Theoretical Studies

### 2.1. Synthesis Method and Deposition of Thin Films

For the synthesis, we used 5 g (0.039 mole) of phtalonitrile mixed with 1.77 g (0.01 mole) of palladium acetate in DMAE with 1 mL of DBU at 140 °C. Then, the mixture was stirred for 2 h at 180 °C. Subsequently, the product was cooled at room temperature and filtered. The obtained solid was finely ground and washed successively with methanol, hot alcohol, and water to remove intermediates and unreacted components. Then, the compound was purified in Bio-Beads using chloroform. The final product obtained was bluish-green in color. The latter was dried in an oven for 1 h and subsequently prepared using the thermal evaporation technique. Evaporation was carried out using resistive heating of about 20 mg of the material in tungsten boat under a vacuum of 5 × 10^−6^ Torr. The boat was heated by passing a high current (100 A). The obtained layers on a clean glass and FTO substrates were homogeneous and had a thickness of approximately 0.1 μm.

### 2.2. Characterization

The absorption spectra were measured using a UV-Vis spectrometer (Shimadzu) with a resolution of 0.1 nm. The optical band gap was estimated using equation (1) and according to the Planck formula as follows [60,61,62,63,64,65]:

(1) $$E_{g}=\Delta E=\frac{hc}{\lambda }$$

where *h* is the Planck constant (6.62617 × 10^−34^ J·s), *c* is the velocity of light (2.9979 × 10^8^ m/s), and *λ* represents the absorption limit wavelength (nm), achieved from the onset of the absorption graph [60,61,62,63,64,65]. The visualization of the surface morphology of the layers was done using atomic force microscopy (AFM, Veeco CP-II) in contact mode with a scanning frequency of 1 Hz and points in Si and Shimadzu-type scanning electron microscopy (SEM, Superscan SSX-550). The synthesis product purity was verified by 1H NMR (Varian XL-200 NMR spectrometer, DMSO d6), elemental analysis (Costech ECS 4010 instrument), and MS and MS-MS spectra (Thermo Quantum Access Mass spectrometer with H-ESI probe conducted in positive ion mode). The analyses were as follows: 1H NMR (DMSO): d, ppm 8.05–8.12(4H, s, broad), 7.71–7.75 (4H, s, broad), 7.4–7.6 (4H, s, broad), and 7.15–7.46(16H, d, broad). Anal. (C_44_H_24_N_16_Pd): C, 59.84; H, 2.74; N, 25.37; Pd, 12.05; found: C, 59.40; H, 3.03; N, 25.82; MALDITOF-MS m/z: calculated 882.14; found + 883.3 (M + 1).

### 2.3. Theoretical Calculations

DFT calculations were performed with Gaussian09w [56,58,66,67] using the functional B3LYP-GD3 with Grimme’s dispersion correction [68] and the SDD basis set [69]. The effect of the DMSO solvent on (PdPc(Im)_4_) was calculated by the SMD model [70]. To study the UV spectra, we used the TD-DFT method on the optimized structure in the DMSO solvent using the same level of theory (B3LYP-GD3/SDD and SMD to simulate the DMSO effect). In addition, we determined the energy of the molecular orbitals and then evaluated the optical gap energy (E_g_) (difference between the HOMO and the LUMO border orbitals and between the molecular orbitals of the main peaks of the spectrum). The PdPc(Im)_4_ structure was optimized using the B3LYP-GD3 function. Frequency calculations were performed to identify the nature of the stationary points.

## 3. Results and Discussion 

### 3.1. The Optimized Molecular Structure

**Figure 1:**
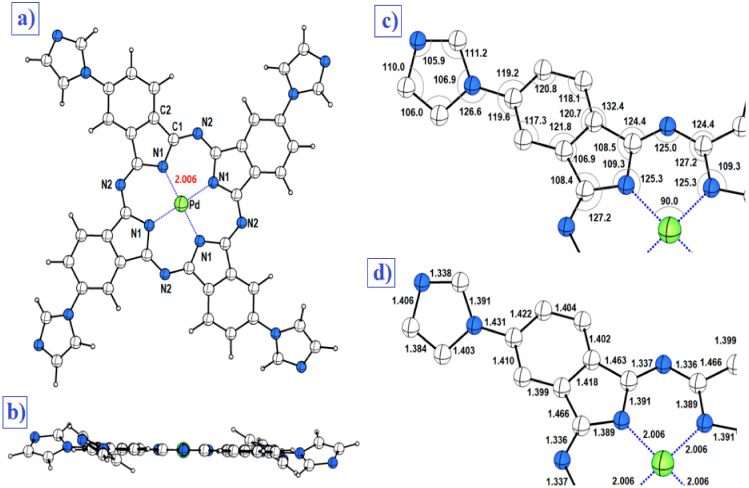
(**a**) Chemical structure of the singlet stat of the neutral PdPc(Im)4 Ci symmetric molecule calculated at the B3LYP-D3/SDD level of theory, taking into account the effect of the DMSO solvent with the SMD model, (**b**) plan structure of the optimized PdPc(Im)4 molecule, (**c**) distances in Å on a quarter (1/4) magnification of the PdPc(Im)4 molecule (all hydrogen atoms have been omitted for clarity of the figure), and (**d**) angles between atoms in degrees represented on a quarter of the molecule for more clarity (all hydrogen atoms have been omitted for clarity of the figure).

Figure 1a depicts the optimized molecular structure of the PdPc(Im)_4_ polymer which shows a square-planar-type configuration with a metal ion at the center (Pd). N atoms are marked in blue; C atoms are in gray. The average Pd–N bond length of the four Pd-N bonds of the optimized structure was 2.006 Å. Figure 1b illustrates the M-plan structure of the PdPc molecule. Figure 1c,d presents distances and angles between atoms in the PdPc(Im)_4_ molecule. Technically speaking, N1 and N2 are the pyrrole and meso positions of nitrogen, respectively. C1 and C2 are the alpha and beta positions of the carbon. The positions, which are of significant interest, are the meso position of the nitrogen that varies with analogous molecules and the position of the nitrogen pyrrole due to the direct bond with the central atom of palladium.

### 3.2. The Frontier Molecular Orbitals (FMOs)

**Figure 2:**
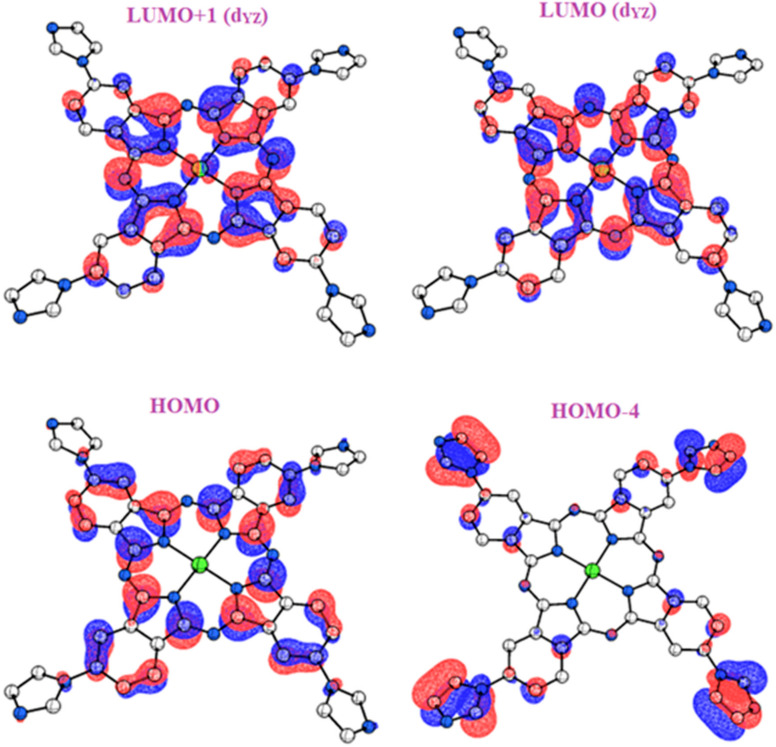
OMs that contribute to UV transitions. Contour plots (contour value = 0.025) of HOMO-n and LUMO + n of PdPc(Im)_4_ calculated at the B3LYP-D3/SDD level of theory in DMSO. LUMO and LUMO + 1 are degenerate.

The LUMO and the HOMO mainly form the frontier molecular orbitals (FMOs). The FMOs are very important for studying the chemical and electrical features of substrates [71]. They impact material properties through the development of their polarities and the abilities for absorbing light. On the other hand, they function as acceptor and donor orbitals [72]. Figure 2 presents the computational study based on the density functional theory (DFT) and time-dependent DFT (TD-DFT). This study was carried out to better understand the geometric and photophysical properties of PdPc(Im)_4_. It describes the HOMO and LUMO electron density, the optimized molecular structures, and the isosurfaces of PdPc(Im)_4_. The apparent plane geometries, the largely delocalized LUMO and HOMO electron densities, and the existence of π and σ orbitals in the FMOs are favorable to the processes of electron migration between these macrocycles. In addition, the related density of state calculations of the PdPc(Im)_4_ was studied in DMSO solution, which presents the virtual and occupied orbitals.

### 3.3. Molecular Electrostatic Potentials

**Figure 3:**
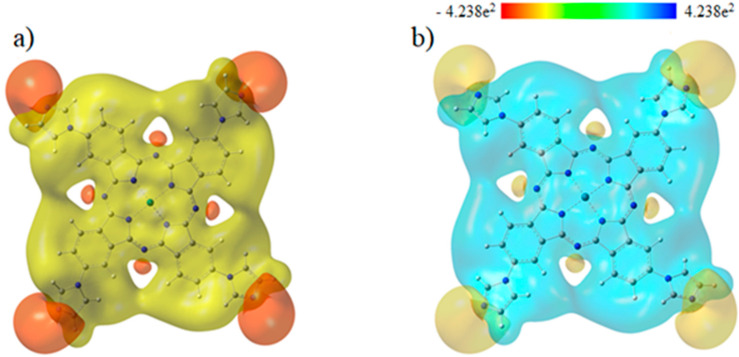
Electrostatic potential surface and contours of PdPc(Im)4: (**a**) from total SCF density (isoval = 0.025); (yellow = positive, orange = negative) and (**b**) mapped with ESP.

**Figure 4:**
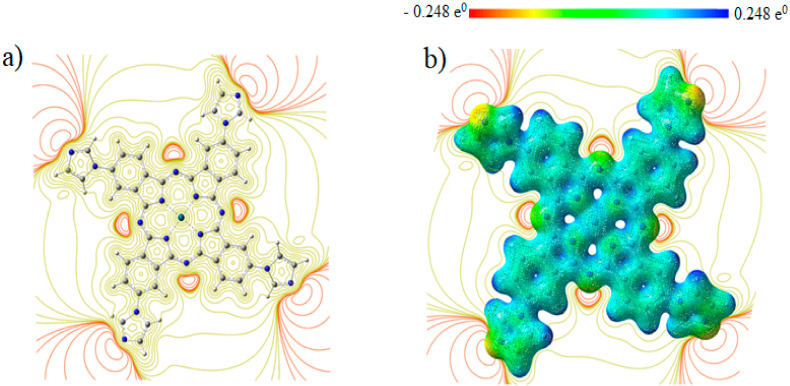
(**a**) Electrostatic potential surface and contours of PdPc(Im)4 from total SCF density (isoval = 0.025) in the plan of molecule (yellow = positive, orange = negative) and (**b**) a cross-section in terms of that mapped with electrostatic potential from total SCF density and contour map.

Molecular electrostatic potentials (MEPs) are very beneficial for studying the relationship between physicochemical features and its molecular structure. This is done by visualization of molecular size and shape, as well as by the charge distribution in the molecule in terms of color calibration [73]. Figure 3 depicts the electrostatic potential surface (EPS) and contours of PdPc(Im)_4_ from the total self-consistent field (SCF) density and mapped with ESP. Figure 4 presents electrostatic potential surface and contours of PdPc(Im)_4_ from the total SCF density in the plan of a molecule and a cross-section in terms of that mapped with electrostatic potential from the total SCF density and contour map on N and Pd atoms. The electrostatic potential surfaces generally render information regarding the stacking of PdPc(Im)_4_ molecules in the sample at the nano-scale. The possible agglomeration in the sample is the H atom, which is owing to a high-energy change in the absorption graph as well as details given by the distribution of electrostatic potential on the molecule.

**Figure 5:**
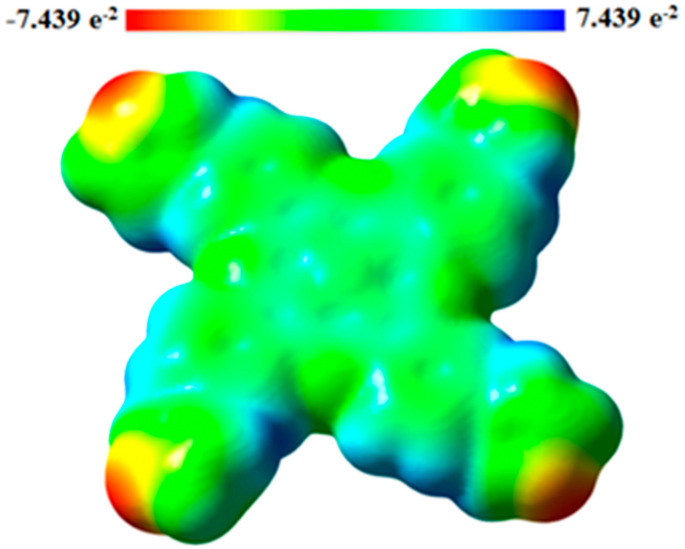
Molecular electrostatic potential (MES) surface for PdPc.

The surface map of the molecular electrostatic potential (MEP) was calculated and is shown in Figure 5. This map shows the existence of four possible sites of electrophilic attack, and it is neutral on the conjugate ring. The region near the central atom of the molecule (Pd) is positive because the carbon atom (C) is surrounded by electropositive atoms. The findings demonstrate that the site including nitrogen atoms are the most reactive site of the PdPc(Im)_4_ molecule. These sites provide details related to the region wherein the compound have intermolecular interplays. The behavior of ESP on the phthalocyanine ring is also related to the magnetic separation and position of chemical shift in C-NMR [74,75].

### 3.4. Optical Absorption Analysis

**Figure 6:**
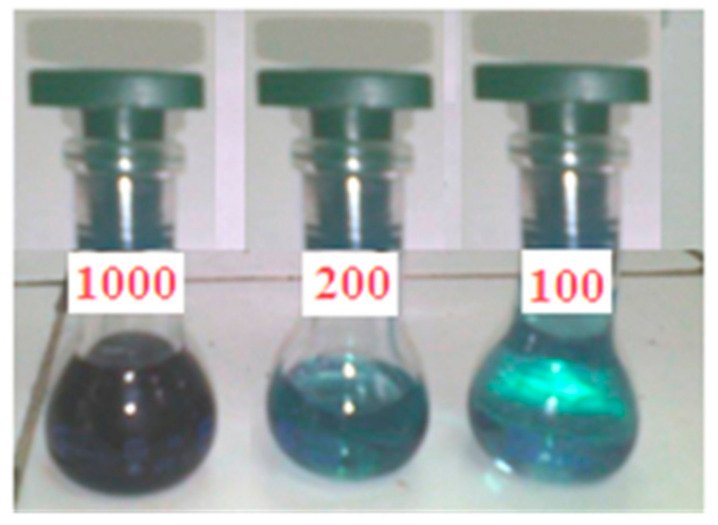
Color of complex at different concentrations (100 mg·L^−1^~1.6 × 10^−4^ M; 200 mg·L^−1^~3.2 × 10^−4^ M; 1000 mg·L^−1^~1.6 × 10^−3^ M).

**Figure 7:**
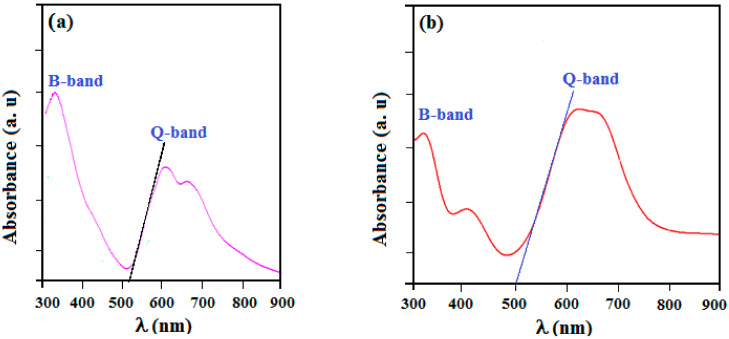
Electronic absorption spectra of PdPc(Im)_4_ in DMSO solution (10^−5^ M) (**a**) and (**b**) thin-film forms.

The optical absorption spectra were made in the wavelength range between 300 and 900 nm using a UV–visible spectrometer. In fact, n→π* and π→π*-type electronic transitions in π-conjugated organic compounds result in a UV-Vis absorption graph [76]. These transitions are usually owing to movements of electrons among boundary molecular orbitals (FMOs). In addition, organic molecules of phthalocyanine and their compounds exhibit optical features due to their cyclic structure. These compounds possess two different types of energy bands including the Q band (a porphyrin band) and the B band (c or Soret band). The peaks observed in the region of the Q band cited in the 610-680 nm range are responsible for the observed green color of this synthesized complex (Figure 6). These transitions can also be attributed to π–π* transitions. Figure 7 shows the UV-Vis absorption spectra for PdPc(Im)_4_ in DMSO solution and thin-film form. For PdPc(Im)_4_ dissolved in DMSO, Figure 7a illustrates a first absorption peak at 350 nm (band B) in the visible spectrum region. A less intense shoulder peak around 610 nm corresponds to the dimer of the phthalocyanine, and a peak at 650 nm to the Q band absorption [77]. Figure 7b depicts the UV-Vis spectrum of PdPc(Im)_4_ thin film. It shows a typical Soret band at around 340 nm, which corresponds to π→π* transition. Furthermore, the band located in the range of 600–680 nm corresponds to Q bands, which was assigned to the dimer of phthalocyanines. (PdPc(Im)_4_) molecules can interact with each other through delocalized π electrons and hydrogen bonds.

**Figure 8:**
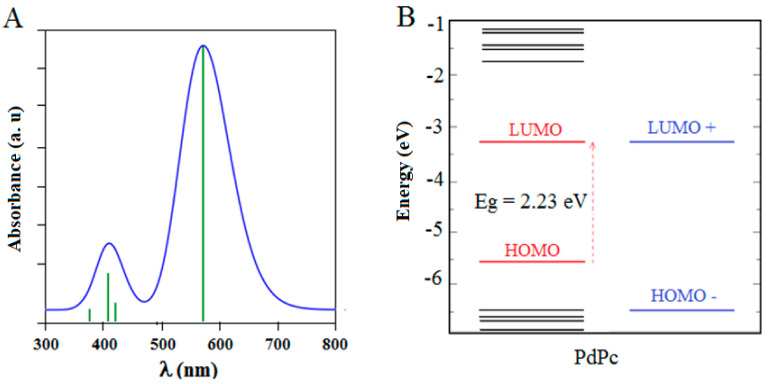
(**A**) Variation of absorbance using DFT and (**B**) PdPc(Im)_4_ molecular orbital energies in eV. The levels to the right show degenerate energies.

**Figure 9:**
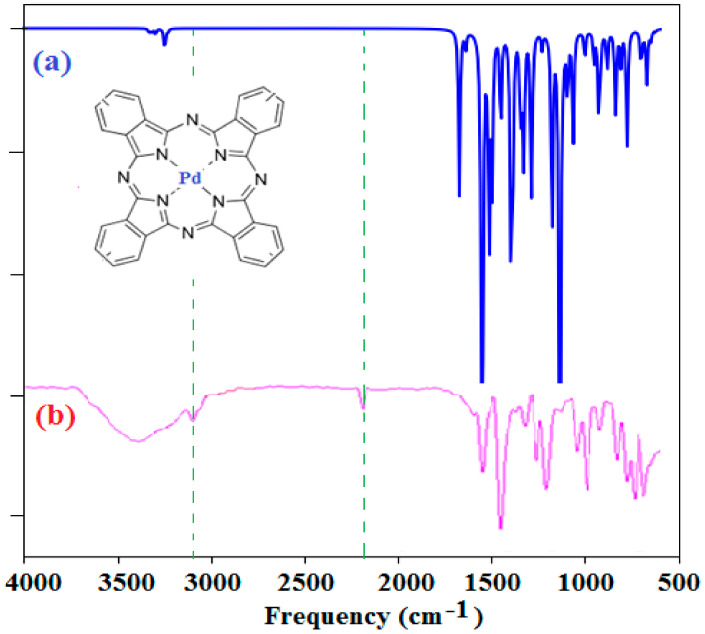
(**a**) Theoretical and (**b**) experimental infrared spectra.

**Figure 10:**
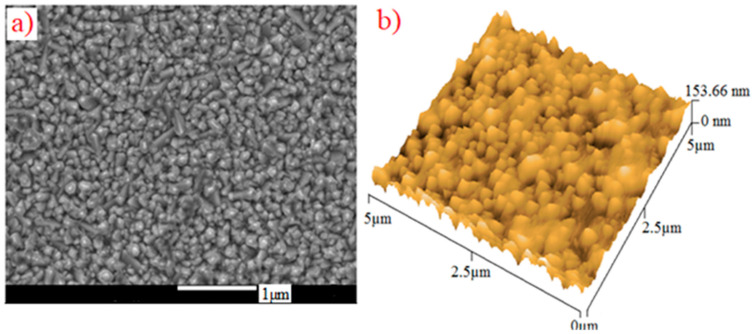
(**a**) SEM analysis and (**b**) three-dimensional AFM analysis of PdPc(Im)4 deposited on FTO by vacuum thermal evaporation technique.

The optical band gaps of the PdPc(Im)_4_ in DMSO solution and thin-film form are also deduced from the figures, which were about 2.41 and 2.48 eV, respectively. The bandgap energy and threshold wavelength were determined using DFT data. The absorption spectrum obtained from the DFT calculations of PdPc(Im)_4_ in the DMSO solvent is presented in Figure 8. The values of energy bandgaps obtained from experimental graphs, theoretical data, and threshold wavelengths are summarized in Table 1. In the Q band, the electronic transition occurred from the electron density centered on the phthalocyanine (HOMO) molecule to the low electron density on the Pd-N bond (LUMO) as demonstrated in Figure 8. The evaluated optical energy was about 2.23 eV. The theoretical optical transition obtained by the TD-DFT calculation is in accordance with that determined experimentally [78]. Recognizable functionality groups of palladium phthalocyanine can be deduced depending on the position of the peak and the intensity of the infrared spectrum. Figure 9 demonstrates the IR spectrum obtained from the DFT method and experimental analysis. A large peak was obtained in the 3100-3500 cm^−1^ range due to the stretch band between O-H and N-H [79,80,81,82,83,84,85]. The peaks are the signals of OH, CH_2_, and C-O [74,75,76,77]. In Figure 9, additional peaks observed at 2200 and 3100 cm^−1^ were assigned to C=N and N-H, which are the characteristic signals of palladium phthalocyanine. For PdPc(Im)_4_ films deposited on FTO, the band gap energy was evaluated based on the study of the absorption graph and the graph described by Tauc [86]. In the literature [87], the obtained band gap energy related to direct transitions in the material is 3.62 eV. The SEM images in Figure 10a shows the morphology of the PdPc(Im)_4_ on the FTO. It is seen that surface was homogenous and composed of grains with a size between 150 and 200 nm. The three-dimensional AFM image in Figure 10b revealed that the PdPc(Im)_4_ layer deposited on FTO caused surface smoothening with spherical grains of different sizes and shapes.

## 4. Conclusions

In summary, the PdPc(Im)_4_ in DMSO solution and thin films deposited on glass and FTO by thermal evaporation were prepared. The UV-Vis technique was employed to establish the optical bandgap of the PdPc(Im)_4_. The optical results demonstrated a band gap of 2.41 eV for the PdPc(Im)_4_ in the DMSO solution and values of 2.48 and 3.62 eV for the thin layers of PdPc(Im)_4_ Pc deposited on glass and FTO, respectively, using a Tauc route. MEP analysis was used to identify electrophilic and nucleophilic sites in the molecule as well as to provide additional information about regions of intermolecular interaction. DFT calculations using the DFT-B3LYP method were also used to calculate the band gap of the PdPc(Im)_4_ molecule, and the achieved bandgap was 2.23 eV, which is close to the experimentally obtained value. The simulated UV-Vis domains are consistent in the shape and position of band B with those of the experimentally obtained results. This study of the PdPc(Im)_4_ band gap is fundamental for various appliances such as organic photovoltaic devices and light-emitting diodes.

## Figures and Tables

**Table 1:** Band gap and threshold wavelengths value for PdPc(Im)_4_ in DMSO solution, PdPc(Im)_4_-thin films, and PdPc(Im)_4_-DFT.

	Q-Band	
Material	Wavelength (nm)	E_g_ (eV)
PdPc(Im)_4_ in DMSO solution	515	2.41
PdPc(Im)_4_-thin films	500	2.48
PdPc(Im)_4_-DFT	490	2.23

## Data Availability

The data used to support the findings of this study are available from the corresponding authors upon request.

## Sample Availability

Further details about the samples investigated in this study are available upon request from the authors.
